# “They should stay at their desk until the work’s done”: a qualitative study examining perceptions of sedentary behaviour in a desk-based occupational setting

**DOI:** 10.1186/s13104-015-1670-2

**Published:** 2015-11-17

**Authors:** Judith A. Cole, Mark A. Tully, Margaret E. Cupples

**Affiliations:** UKCRC Centre of Excellence for Public Health (NI), Centre for Public Health, Department of General Practice, School of Medicine, Dentistry and Biomedical Sciences, Queen’s University Belfast, Dunluce Health Centre, 1 Dunluce Avenue, Belfast, BT9 7HR Northern Ireland; UKCRC Centre of Excellence for Public Health (NI), Centre for Public Health, School of Medicine, Dentistry and Biomedical Sciences, Queen’s University Belfast, Room 02020, Institute of Clinical Science B, Royal Victoria Hospital, Grosvenor Road, Belfast, BT12 6BJ Northern Ireland

**Keywords:** Sedentary, Workplace, Sitting time, Behaviour change, Incentives, Barriers

## Abstract

**Background:**

Workplace sedentary behaviour is a priority target for health promotion. However, little is known about how to effect change. We aimed to explore desk-based office workers’ perceptions of factors that influenced sedentary behaviour at work and to explore the feasibility of using a novel mobile phone application to track their behaviours.

**Methods:**

We invited office employees (n = 12) and managers (n = 2) in a software engineering company to participate in semi-structured interviews to explore perceived barriers and facilitators affecting workplace sedentary behaviour. We assessed participants’ sedentary behaviours using an accelerometer before and after they used a mobile phone application to record their activities at self-selected time intervals daily for 2 weeks. Interviews were analysed using a thematic framework.

**Results:**

Software engineers (5 employees; 2 managers) were interviewed; 13 tested the mobile phone application; 8 returned feedback. Major barriers to reducing workplace sedentary behaviour included the pressure of ‘getting the job done’, the nature of their work requiring sitting at a computer, personal preferences for the use of time at and after work, and a lack of facilities, such as a canteen, to encourage moving from their desks. Facilitators for reduced sedentariness included having a definite reason to leave their desks, social interaction and relief of physical and mental symptoms of prolonged sitting. The findings were similar for participants with different levels of overall physical activity. Valid accelerometer data were tracked for four participants: all reduced their sedentary behaviour. Participants stated that recording data using the phone application added to their day’s work but the extent to which individuals perceived this as a burden varied and was counter-balanced by its perceived value in increasing awareness of sedentary behaviour. Individuals expressed a wish for flexibility in its configuration.

**Conclusions:**

These findings indicate that employers’ and employees’ perceptions of the cultural context and physical environment of their work, as well as personal factors, must be considered in attempting to effect changes that reduce workplace sedentary behaviour. Further research should investigate appropriate individually tailored approaches to this challenge, using a framework of behaviour change theory which takes account of specific work practices, preferences and settings.

## Background

‘Too much sitting’ has been proposed as a separate concept to ‘too little exercise’ [1]. Sedentary behaviour, as distinct from a lack of physical activity, has emerged in recent years as a risk factor for adverse health consequences [2, 3]. It is described as activity which does not increase energy use above resting (for example, sleeping or sitting) and the importance of distinguishing sedentary behaviour from light physical activity (for example, slow walking or cooking food), has been emphasised [4].

Systematic reviews [3, 5] have reported evidence which suggests that there is a link between sitting time and increased risk of cancer, cardiovascular disease and mental disorders. Much recent research has focused on leisure time sedentary behaviour, particularly television viewing [6, 7]. However, occupational sitting is the major cause of sedentary behaviour in many industrialised countries [8] and the workplace has been recognised as a priority setting for health promotion by the World Health Organisation (WHO) [9]. NICE guidelines [10] outlined recommendations for encouraging employees to be more physically active and less sedentary in their workplace. Breaking up periods of prolonged sitting with bouts of light-intensity activity has beneficial metabolic effects [11] but studies which have tested interventions aimed to reduce sitting time in workplaces have shown variable success.

For example, Pronk et al. [12] designed a 4-week intervention in which the study group received an adjustable desk which supported a computer, allowing employees to stand or sit at work. Participants were sent text messages at random times during the day to ask if they were sitting, standing or walking at that moment; their average sitting time was reduced by 224 % (66 min per day) and they reported improvements in upper back and neck pain and mood. However, a 2-year randomised controlled trial [13] of an interactive internet-delivered workplace health promotion programme, delivering tailored advice in response to online tracking of lifestyles, found no evidence of benefit compared to a single physical health check with advice and tailored feedback. Low levels of use of the internet programmes were reported and it was suggested that behaviour change needs both individual and environmental influences, with possible workplace organizational change.

Bort-Roig et al. [14] tested an internet-based intervention aimed to encourage less sitting, by integrating more activity into work tasks. Daily step counts were recorded using a pedometer and diary, and a website offered progress reports and motivational tips. Overall, 67 % increased their step count while 60 % decreased occupational sitting: ‘screen based work’ was identified as the most significant barrier to change. Smartphone technology has been used to change sedentary behaviour in other settings [15, 16] but its potential use in helping to reduce occupational sedentary behaviour among young professionals has not been reported.

Proper et al. [17] and Thorp et al. [5] concluded that more research was needed regarding interventions to reduce sedentary behaviours. Renton et al. [18] and Taylor et al. [19] called for more research on participant (employee and/or employer) views of the feasibility of reducing sedentary behaviour at work. Wilmot et al. [3] identified that little is known about how best to change sedentary behavior and Owen et al. [20] commented that feasibility studies were needed to examine the acceptability of interrupting and reducing occupational sedentary time. Further research is needed to help determine appropriate theoretical frameworks and environmental change to promote less sitting.

The current study sought to target this gap in the evidence by exploring the perceptions of young professional office workers and their managers regarding factors that limited or encouraged sedentary behaviour at work and to examine the feasibility of using a novel mobile phone application to record the detail and duration of their activities during the day.

## Methods

### Phase 1

All employees (n = 12) and managers (n = 2) of a small software engineering company, located in a United Kingdom city centre, surrounded by other small businesses and close to areas of socio-economically deprived inner city housing, were invited to participate. Employees were asked to return a confidential sealed reply slip to the researcher.

The researcher met those who agreed to take part in interviews at their office to explain the proposed research and ask for their written consent. Interviews were semi-structured and audio tape recorded, lasted between 20 and 50 min and covered topics such as participants’ perceived barriers and facilitators affecting sedentary behaviour in the workplace, their current behaviour and opportunities to reduce their sitting time.

Examples of open-ended questions asked during the interview are:

So in terms of the office then, what would your views be about sitting for long periods?

How do you feel when you get up from the desk and stand or walk somewhere?

How would you describe the office culture here?

So what would prevent you from taking more breaks then?

At the end of the interview participants were asked to wear an accelerometer for 1 week and a date was agreed for the researcher to collect the device. Verbal and written information was given to participants regarding access to online versions of validated questionnaires to assess their overall levels of physical activity behaviour (Global Physical Activity Questionnaire, GPAQ) [21] and self-efficacy [22], i.e., their belief in their own ability to complete certain tasks and goals [23]. Participants were invited to complete the questionnaires confidentially, using a unique ID and password which was created for each participant. Participants who did not return online questionnaires were offered paper copies to complete.

### Phase 2

The participants who completed interviews (seven), wore an accelerometer and returned questionnaires were invited to take part in the second phase of the study; an additional six employees agreed to take part at this stage.

They were asked to wear an accelerometer throughout each day for 2 weeks and were invited to test the use of a mobile phone tool, a downloadable application in which participants could record and review their activities throughout the day. This application was configured to allow tracking of pre-set categories of activity, with choices of labels and colours and intervals of 5 min duration. It allowed creation of templates for rapid entry of longer duration intervals of activities and free text entry to describe what was being done at different times but did not allow individuals to adapt its configuration.

The researcher visited the office to deliver and give instructions concerning use of the accelerometer and to inform participants about the use of the application and about the end of study questionnaire which related to the feasibility and usefulness of the mobile phone tool.

### Data management and analyses

All interviews were transcribed verbatim and the NVivo computer software programme (version 9, QSR International Pty Ltd.) was used in analysis. Two researchers (JC and MC) reviewed the transcripts using a thematic analysis framework. Within each interview transcript, researchers identified salient words, phrases and sentences about barriers and facilitators to sedentary behaviour in the workplace and assigned them codes which were then grouped within the pre-identified themes and new sub-themes were created. Major and recurrent themes were discussed and validated through discussion among the research team.

The GPAQ responses [21] were used to place participants in one of three categories, assessing their total physical activity levels as high [>3000 Metabolic Equivalents of Task (MET) min/week], moderate (1500–3000 MET min/week) or low (<1500 MET min/week).

The accelerometers [Actigraph GT3X tri-axial accelerometers (Actigraph Inc, Fl)] measured physical activity and time in sedentary behaviours. Sedentary time, representing periods of very low intensity activity, was classified as activities <1.5METs, calculated as time spent below 100 counts per minute; time spent in moderate or vigorous physical activity (MVPA) was classified as time spent in activities greater than 2691 counts per minute, and classified using Actilife v6.7.1 software. Only time spent in normal working hours (9 a.m.–5 p.m.) was analysed. The lengths of bouts of sedentary behaviour were automatically classified by the accelerometer software. A bout was deemed to have ended when a movement above the pre-set threshold was undertaken. Patterns of sedentary behaviour during office hours were analysed for all participants. Accelerometer data for participants who took part in both phases of the study were compared before and after the use of the tool, to examine possible change in behaviour within individuals.

### Ethics approval

This study was granted approval by the Queen’s University Research Ethics Committee on 24th April 2013, Reference Number 13/10v2.

## Results

Of the seven (five employees, two managers) participants who completed the interview all were software engineers; four completed an online GPAQ questionnaire and two completed paper versions. GPAQ data indicated that participants’ levels of physical activity varied widely (from 30 to 450 min per day and from 120 to 5880 MET min/week); their sedentary behaviour time varied from 6 to 15 h (Table 1). With regard to assessment of self-efficacy, participants were asked to consider six different situations and state how confident they were that they could reduce sedentary behaviour in each (Table 2). Of the six respondents who completed the self-efficacy questionnaire, all completed paper versions. No-one was very confident that they could reduce their sedentary behaviour when they were tired; all had some level of confidence that they could reduce it in the office environment but they all were very or extremely confident in doing so when away from work.

**Table 1:** Time spent in physical activity and sedentary behaviour, assessed by GPAQ

Participant ID number	PA per day (mins)	MET mins per wk	Level of total PA	Sedentary time per day (hrs)
2	30	120	Low	12–15
3	450	5880	High	6–9
4	210	3960	High	6–9
5	150	720	Low	12
6	135	1260	Low	6
7	150	1920	Moderate	9–12

**Table 2:** Numbers of participants responding to self-efficacy questionnaire

Stem question: how confident are you that you could reduce sedentary behaviour when you…	Not at all confident	Slightly confident	Moderately confident	Very confident	Extremely confident
Are tired	1	1	4	0	0
Are busy at work	2	2	1	1	0
Feel you don’t have time	1	4	1	0	0
Are in the office environment	0	1	2	2	1
Are in a bad mood	0	1	3	0	2
Are resting away from work	0	0	0	3	3

Interview analysis identified themes and sub-themes, related to barriers and facilitators affecting sedentary behaviour within the office environment. The findings are supported, as presented below, by participants’ quotations, identified in respect of their sex (Male/Female), employee (Employee) or manager (Manager) status and, where available, level of physical activity (PA), (assessed by GPAQ, as high, moderate or low).

### Barriers to reducing sedentary behaviour

#### Nature of the job

The majority of participants reported perceptions that sitting, rather than standing, was appropriate to their work with computers. They considered that standing might cause discomfort and affect their work performance adversely. Further, they perceived that time spent sitting was a reflection of the volume of their work. These perceptions did not appear to be related to an individual’s overall level of physical activity.

You’ve got so much to do you don’t want to think about the tiredness of your legs if you were standing. (P3, Female, Employee, High PA).

In the software environment, you need to sit down, because you need to concentrate. (P5, Male, Employee, Light PA).

To work you have to be at the computer. (P4, Male, Employee, High PA).

One employee reported that he had heard of ‘standing desks’ and gave an example which showed that productivity was not decreased but acknowledged that sitting was a habit which had formed over many years in the job.

It’s the nature of programming to focus for long periods of time, it’s the pattern you fall into … just habit. (P6, Male, Employee, Low PA).

Employees’ comments suggested that they perceived that sedentary behaviour was influenced by a pressure of the amount of work which they needed to deliver: they perceived that their output might fall if they interrupted their workflow by leaving their desk and this pressure was accentuated by a feeling of inter-dependency on others’ work efforts.

You feel it’s (getting up from the desk) going to break up the flow of the piece of work you’re doing, that’s maybe why you wouldn’t leave the desk. (P7, Male, Employee, Moderate PA).

… depends on how much other people are relying on you. … it’s kind of like a cycle …, you get pulled back into the work, due to someone else’s deadline. (P3, Female, Employee, High PA).

These perceptions of a culture of expectation that the job required that people would be sedentary when at work were shared by those who had a management role. Indeed, managers expressed this view in very candid language: the company needed work done to survive, and for work to be accomplished employees needed to be sitting at their computers:

We just need people who work all hours to get stuff done … I’m a great believer that they should stay at their desk and do it. (P1, Male, Manager).

Traditionally offices have cubes and you put the software engineers in there… until they’ve finished, limit the amount of distractions so that they get stuff done. (P1, Male, Manager).

It was also suggested that it was important to work when sitting at a desk because of what others in an open-plan office might think, although employees did not recognize any sense of an imposed restriction in their activity.

“I do think there is a degree of the kind of peer social element to whether or not you’re seen to be working.” (P2, Male, Manager, Low PA).

“I think that’s a different company you’re talking about. That’s not here … nobody is forced or asked to or expected to work long hours here.” (P6, Male, Employee, Low PA).

Schemes or ideas to promote less sedentary time were perceived as a luxurious extra, peripheral to core business, rather than a factor which might contribute to the health or overall productivity of the company:

… all this stuff is luxury, I’d love it if it were otherwise … (P1, Male, Manager).

#### Preferred time for physical activity

Comments indicated that people preferred to focus on work and stay sedentary during their working hours, targeting their time away from work as a time for physical activity. This was despite acknowledging that their employers did not restrict their choice of being more active during the working day. The participants recognized that they had autonomy in decisions regarding the time they spent being sedentary or physically active during working hours. Their comments reflected a sense that they had made considered decisions.

I usually work 8 h, it’s 8½ work day, with an hour lunch, but I usually only ever really take a half an hour, so I can leave a wee bit early to get to the gym and do my fitness after work. But there’s no restrictions, it’s just my choice really … people go for walks … but that’s not really for me–with my lunch I want to relax, I want to maybe read, and do my exercise at another time, in my own space. I don’t want to come back to work being sweaty from my power walk. (P3, Female, Employee, High PA).

Other participants also made decisions to remain sedentary during working hours at times when they were not working: they enjoyed and prioritized relaxation activities which were linked with sedentary behavior rather physical activity.

Other reasons for prolonged sitting would be actually internet browsing, so, that’s a main thing, people who entertain themselves when they have a break by continuing to work at a computer. (P2, Male, Manager, Low PA).

#### Facilities within the work environment

Comments were made regarding the physical environment in which they worked, and how that this could be changed to make it more conducive to reducing sedentary behavior.

It’s a pity that we don’t have a canteen … a lot of office workers will sit at their desk and eat their lunch… I do think they need to have more social areas, away, completely away, in another room from the work space … because you get pulled back (to work jobs). (P3, Female, Employee, High PA).There’s nowhere to go here … there’s no canteen or anything else. (P4, Male, Employee, High PA).

### Facilitators for reducing sedentary behaviour

#### Purpose in movement

Participants commented that having a purpose to get away from their desk prompted them to become physically active. Good weather also provided an incentive to leave their desk and work and go outside.

If I need to get up and do something I will, speak to colleagues or even go to the printers, to get stationery, things like that. (P3, Female, Employee, High PA).

“(Making) coffee helps. When the weather’s good, I go for a walk.” (P5, Male, Employee, Low PA).

#### Relief of symptoms

Comments indicated that participants had experienced physical and mental effects of prolonged sitting and both employees and managers realized that these could be relieved by taking short breaks.

(Getting up more) would make me feel more refreshed … typically I would be getting up in order to discuss with people, you know, and its interaction, so, you know, the more of that the easier the day goes. (P2, Male, Manager, Low PA).

A male employee said that he had experienced back problems from sitting for long periods and that getting up from his chair relieved these “to some extent”. However, this incentive to reduce his sedentary behavior was tempered by his sense of responsibility to the company, which was his priority. He commented that he did not take more breaks because “I get paid for seven and a half hours (work) per day”.

#### Peer support

In contrast to comments indicating how the work environment pressed employees to be sedentary, other comments showed that the office environment was not restrictive to taking breaks and that a degree of flexibility in this regard was appreciated. Employees were conscious of how their work colleagues spent their time in the office and they reported various situations at work which encouraged social interaction and physical activity.

We’re pretty good here with regard to flexibility, if I need to pop out or I need to go the shops I’ll go out for 5 min. Office morale here is quite good, because they’re lenient … you get up, just so long as everybody does their best. (P3, Female, Employee, High PA).

The chance to break away from the desk for even 10 min was appreciated and found to be helpful. Interestingly also, despite others’ comments, one participant had identified a space within the work building to which he could go from his desk.

I go downstairs to the pantry there at about 12 noon … we start talking to people about different things, and that is relaxing, you only take about 10 or 15 min and then you come back and start work again. (P5, Male, Employee, Low PA).

Even taking time to meet with colleagues and talk about work in a different area of the office—and while standing together—was an opportunity to relieve the long sitting periods:

Sometimes we come here and have whiteboard sessions and everyone has a chance to stand up and write something on the board, discussion, it’s better than sitting. (P5, Male, Employee, Low PA).

### Feasibilty of using mobile phone application

#### Participants’ sedentary behaviour

Valid baseline data were available for 5 participants (Table 3). The majority of time during office hours was spent in very light intensity sedentary behaviour (401/480 min = 83 %). Overall, 4 of the 5 participants took more than 30 min of moderate or vigorous physical activity daily during these hours.

**Table 3:** Accelerometer data: time in sedentary and active behaviours during office hours

	Baseline mean (range) (n = 5)	Follow-up mean (range) (n = 11)
Sedentary (mean daily mins)	401.90 (377.30–421.90)	384.19 (267.00–450.10)
Physical activity (mean daily mins in moderate/vigorous PA)	36.47 (27.39–48.08)	22.53 (12.78–29.86)
Steps per day	3134.46 (2430.75–3887.33)	2379.76 (425.46–3848.20)

Data were available for 11 participants at the follow-up time point (Table 3). The mean time spent in sedentary behaviour, minutes of MVPA and steps counts per day were lower in this larger group. Time spent sedentary declined in all four individuals for whom data were suitable for analysis at both time points; the maximum length of a single bout of sitting fell in three of these participants (Table 4) and PA (mins of moderate or vigorous activity) decreased in all four.

**Table 4:** Time spent in sedentary behaviour and physical activity at baseline and follow-up

ID	Daily average mins in sedentary behaviour			Maximum length (mins) of sedentary bouts			Mean mins of moderate or vigorous PA per day			Mean steps per day		
	Baseline	Follow-up	% change	Baseline	Follow-up	% change	Baseline	Follow-up	% change	Baseline	Follow-up	% change
2	378.4	267	−29	22.9	20.3	−11	39.8	20.8	−48	3230	3848	19
4	420.1	398.9	−5	17.9	42.5	137	33.9	20.2	−41	3039	2259	−26
5	421.0	317.2	−25	14.7	14.2	−3	27.4	22.9	−16	2431	2397	−1
7	411.8	364.6	−11	28.3	23.5	−17	33.2	27.0	−19	3086	3315	7
Mean	406.8	327.7	−19	18.5	25.7	39	33.7	21.3	−36	2890	2835	−2

#### Experience of using mobile phone application

Eight participants completed the study exit questionnaire, which sought information on their use, experiences and views of the mobile phone application. Individuals varied in their frequency of data entry: some used the tool once daily or less (n = 3), some 2–4 times daily (n = 2) and others did so >4 times/day (n = 3).

Overall, whilst a somewhat negative view of the usefulness of the tool in recording their daily activities was expressed, since the task of entering data added to their day’s work, some participants stated that entering data about ‘how you spend your time’ increased their awareness of time spent in various activities. Free text comments included that ‘it was useful in reducing sedentary behaviour’ and that ‘seeing the red bar growing (reflecting sitting) had an impact’. Some reported that it was ‘simple to use’, ‘easy to track, modify, add data, input time period’ but others reported that it was ‘cumbersome’, ‘editing was difficult’ and it was ‘not intuitive’.

Several gave detailed feedback regarding how they would wish to change the software configuration in order to tailor its use to their personal preferences for data recording and presentation of information.

## Discussion

### Summary of findings

Qualitative analysis of participants’ interviews revealed that major barriers to reducing occupational sedentary behaviour were the nature of their job, with a perceived pressure to spend most of their working day sitting at a computer; a preferred time for physical activity, with preference to plan to exercise after working hours; and the physical work environment, with a lack of facilities, such as a canteen, to encourage moving away from their desks. Clear themes also emerged for facilitators: having a definite purpose to move from their desk, relief of physical or mental symptoms of prolonged sitting, and peer support, with a relaxed working environment encouraging social interaction.

#### Enjoyment and social interaction

Few previous studies have examined the barriers and facilitators related to reducing sedentary time in the workplace. However, in keeping with our findings that a reduction in sedentary behaviour at work is likely to be facilitated by opportunities for greater social interaction, Taylor et al. [19] found that a 1-year intervention which encouraged employees to participate in one 15-minute session of physical activity per day had benefits which included enjoyment of more social interactions with colleagues and reduced stress. Their study was conducted in a US city with 82 employees from 5 workplaces, including a law firm, hospital, education agency, city health department and court reporting firm. Their participants reported increased health awareness but also identified a need for more variety in the activities offered and, in concordance with our participants, identified the importance of having management support for taking physical activity.

#### Context of the work environment

While our participants spoke of an office culture that allowed short breaks without fear of criticism, they also highlighted an over-riding priority of getting the ‘stuff’ done and an inter-dependency with others which needed them to be at their desks. The context of difficult economic times and a relatively small software engineering firm, in which employees are under pressure to solve problems as quickly as possible is a significant factor in planning how to support behaviour change to reduce sitting time. Our self-efficacy questionnaire revealed that most participants had greater confidence that they could reduce their time spent sedentary when they were away from, rather than at, work. Interestingly, ‘screen based work’ was identified by Bort-Roig et al. [14] as being the most significant barrier limiting the uptake of strategies to reduce sedentary time at work.

Consideration of such context emphasises the importance of applying theoretical frameworks to the planning of interventions which take account not only of personal factors but also of the physical and mental environment of the workplace. Michie at al’s behaviour change wheel [24] recognises the contribution of the individuals’ capability and motivation to achieve behaviour change as well as their opportunity to do so. Thus there is a need to consider how to support organisations in their provision of opportunities to reduce sitting time at work rather than introduce legislation which requires implementation of this as a directive. The perceived potential impact of such change in working practices on a business’s economic viability supports the suggestion [18] that financial incentives for companies to set up initiatives to reduce workplace sedentary behaviour should be considered.

#### Personal factors

Of interest is the difference found in personal approaches to reducing sitting time at work. A number of participants in our study expressed a preference for leaving any physical activity until the evening time, outside the workplace. Furthermore, while they felt pressure to sit to work, several also preferred to use lunch times or other breaks for sedentary relaxation rather than for physical activity. Bort-Roig et al. [14] found that time pressures and cultural norms in offices were important in shaping work practices such as ‘walk-talk meetings’ and ‘lunch time walking groups’.

Several employees in our study identified that there were limited facilities in their workplace to encourage moving away from their desks, reporting that there was no canteen, but one individual had found a nearby pantry that allowed social interaction with workers from neighbouring businesses and a purpose for some physical activity. We suggest this illustrates how individuals with different attitudes, beliefs or priorities may perceive and use opportunities differently and reflects the significance of individual motivation within approaches to changing behaviour [24].

Our participants reported wide variation in the use of a mobile phone application that was designed to support them in tracking their behaviour and thus increase their awareness of time spent sitting. Their varied views regarding the ease of use and appropriateness of configuration of that tool also illustrates the need to consider individuals’ preferences within corporate strategies.

### Strengths and limitations of the study

Our study presents novel insights into the perspectives of both employees and managers regarding sedentary behaviour at work, reflecting the influences of working in an extremely high pressure environment during a time of economic recession. There were practical difficulties in conducting the fieldwork due to changes in employees’ schedules, time constraints and a need to avoid impingement upon the company’s work flow. Fewer participants than planned participated in the interviews but our interviewees included a range of differing individual characteristics and we identified that data saturation was achieved, with no new issues being identified in later interviews.

Previous work has reported that total daily sitting time was negatively associated with age and overall physical activity and positively associated with BMI [25]. We did not present information about the age or BMI of our participants as this would have compromised their identity but we did not observe a similar association and, given the small numbers included in our study, statistical analysis of data would not have been appropriate. However, the quotations we have presented illustrate how there was concordance in the views expressed by people of widely varying levels of overall physical activity.

The small number of participants whose data allowed tracking of change for individuals following use of the phone tool to record their daily activities limits our interpretation of the changes observed. Time constraints prevented us gathering further follow-up information to explain, for example, why there was a 137 % increase in the longest bout of sedentary behaviour in one participant or the apparently contradictory decline in both sedentary and moderate or vigorous physical activity. We may conclude that individuals had spent more time in light intensity activity but further interviews and more in-depth data at the follow-up time point would inform future strategies involving the use of a mobile phone tool in seeking to reduce sedentary behaviour in a computer or desk-based workplace.

The mobile phone application which was offered to study participants did not allow individuals to adapt its configuration: participants were not offered the option of using online services which would have enabled remote configuration of the application. We did not explore, within this study, how individuals might have chosen to tailor it to their preferred style or ‘habit’ of recording data. However, given the individual variation noted in its perceived ‘user friendliness’, we suggest that future work should examine participants’ personal preferences regarding the presentation and function of applications which are used to track daily activities.

### Implications for research and practice

Our findings highlight the immense challenge of promoting less sitting time within high pressure work environments. They support Owen et al.'s [26] statement that a behaviour- and context-specific approach is required, within an ecological model of sedentary behaviour in which settings and their characteristics determine behaviour. Thus, to address our participants’ perspectives that it is necessary to sit at a desk in order to perform computer programming work, important aspects of the physical setting should be considered, including norms such as having easily available chairs at every computer and of sitting during meetings [26].

Also, we found that social support and the behaviour of peers was a strong influence on participants’ sedentary behaviour and their perception of their ability to change. This has significant implications, in keeping with the principles of a social ecological model of behaviour change, for the need to show recognition of the importance of social and cultural influences within the work environment on people’s behaviour. Attention should be given to individuals’ needs for social support in behaviour change, including their perceived self-efficacy.

However, given the variation in views regarding the feasibility of using the mobile phone application which was tested and the expression of personal preferences for how participants spend time at work, in respect of using non-work time for activities that include sitting, there is a need to consider the priority given to personal preferences, such as within behaviour choice theory [27]. The application of this theory to behaviour change may be considered so that times during the working day are identified, in collaborative discussion with employees, as suitable for non-sedentary activity and that companies offer rewards for engaging in such.

There is potential value in using a mobile phone application to record the duration of various activities, including sitting, during the working day and thereby heighten awareness of, and reduce, sedentary behaviour. However, in order to minimize any perception of its use adding to the daily workload, refinement of its design should explore the value of enabling its adaptation to individuals’ varying preferences for how and what detail of activity data are recorded.

## Conclusions

The barriers and facilitators relating to workplace sedentary behaviour, as identified in our study of a small software computer company, show that any effort to impact upon unhealthy office lifestyle should incorporate cultural, physical and personal factors. While interventions to reduce sedentary behaviour should be tailored to individual workplaces, and incorporate a multilevel strategy as detailed in the ecological model of behaviour change [28], more research investigating different approaches is required to ascertain which principles and components are most likely to be effective. More research needs to be carried out into ways to reduce sedentary behaviour at work and to assure employers of the benefits of less sitting time with no loss of productivity.

## Abbreviations

NICE: National Institute for Health and Care Excellence
GPAQ: Global Physical Activity Questionnaire
MET: Metabolic Equivalents of Task
PA: physical activity
P: participant
MVP: moderate or vigorous physical activity

## References

[1] Owen N, Healy GN, Matthews CE, Dunstan D (2010). Too much sitting: the population-health science of sedentary behaviour. Exerc Sport Sci Rev.

[2] Hogan CL, Catalino LI, Mata J, Fredrickson BL (2015). Beyond emotional benefits: physical activity and sedentary behaviour affect psychosocial resources through emotions. Psychol Health.

[3] Wilmot EG, Edwardson CL, Achana FA, Davies MJ, Gorely T, Gray LJ, Khunti K, Yates T, Biddle SJH (2012). Sedentary time in adults and the association with diabetes, cardiovascular disease and death: systematic review and meta-analysis. Diabetologia.

[4] Pate RR, O’Neill JR, Lobello F (2008). The evolving definition of sedentary. Exerc Sport Sci Rev.

[5] Thorp AA, Owen N, Neuhaus M, Dunstan DW (2011). Sedentary behaviors and subsequent health outcomes in adults. Am J Prev Med.

[6] Sugiyama T, Healy GN, Dunstan DW, Salmon J, Owen N (2008). Is television viewing time a marker of a broader pattern of sedentary behaviour?. Ann of Behav Med.

[7] Clark BK, Sugiyama T, Healy GN, Salmon J, Dunstan DW, Owen N (2009). Validity and reliability of measures of television viewing time and other non-occupational sedentary behaviour of adults: a review. Obes Rev.

[8] British Heart Foundation National Centre (BHFNC) for Physical Activity and Health. 2012. Sedentary behaviour evidence briefing. http://www.bhfactive.org.uk/files/525/sedentary_evidence_briefing.pdf. Accessed 3 Jun 2013.

[9] World Health Organisation (WHO). http://www.who.int/mediacentre/factsheets/fs311/en/index.html. Accessed 3 Jun 2013.

[10] National Institute for Health and Clinical Excellence public health guidance 13: 2008. Promoting physical activity in the workplace. http://www.guidance.nice.org.uk/ph13. Accessed 3 Jun 2013.

[11] Dunstan DW, Kingwell BA, Larsen R, Healy GN, Cerin E, Hamilton MT, Shaw JE, Bertovic DA, Zimmet PZ, Salmon J, Owen N (2012). Breaking up prolonged sitting reduces postprandial glucose and insulin responses. Diabetes Care.

[12] Pronk NP, Katz AS, Lowry M, Payfer JR (2012). Reducing occupational sitting time and improving worker health: the take-a-stand project, 2011. Preventing Chronic Dis.

[13] Robroek SJW, Polinder S, Bredt FJ, Burdorf A (2012). Cost effectiveness of a long term internet-delivered worksite health promotion programme on physical activity and nutrition: a cluster randomised controlled trial. Health Educ Res.

[14] Bort-Roig J, Martin M, Puig-Ribera A, Gonzalez-Suarez AM, Martinez-Lemos I, Martori JC, Gilson N (2014). Uptake and factors that influence the use of ‘sit less, move more’ occupational intervention strategies in Spanish office employees. Int J Behav Nutr Phys Act.

[15] Kirwan M, Duncan MJ, Vandelanotte C, Mummery WK (2012). Using smartphone technology to monitor physical activity in the 10,000 steps program: a matched case–control trial. J Med Internet Res.

[16] Bond DS, Thomas JG, Raynor HA, Moon J, Sieling J, Trautvetter J, Leblond T, Wing RR (2014). B-Mobile—a smartphone-based intervention to reduce sedentary time in overweight/obese individuals: a within-subjects experimental trial. PLoS One.

[17] Proper KI, Singh AS, van Mechelen W, Chinapaw MJ (2011). Sedentary behaviours and health outcomes among adults: a systematic review of prospective studies. Am J Prev Med.

[18] Renton SJ, Lightfoot NE, Maar MA (2011). Physical activity promotion in call centres: employer’s perspective. Health Educ Res.

[19] Taylor WC, King KE, Shegog R, Paxton RJ, Evans-Hudnall GL, Rempel DM, Chen V, Yancey AK (2013). Booster Breaks in the workplace: participants’ perspectives on health-promoting work breaks. Health Educ Res.

[20] Owen N (2012). Sedentary behaviour: understanding and influencing adults’ prolonged sitting time. Prev Med.

[21] World Health Organization (2005). WHO steps surveillance manual: the WHO stepwise approach to Chronic disease risk factor surveillance.

[22] Marcus BH, Forsythe L (2009). Motivating people to be physically active.

[23] Bandura A (1977). Self efficacy: toward a unifying theory of behavioural change. Psychol Rev.

[24] Michie S, van Stralen MM, West R (2011). The behaviour change wheel: a new method for characterising and designing behaviour change interventions. Implement Sci.

[25] Bennie JA, Pedisic Z, Timperio A, Crawford D, Dunstan D, Bauman A, van Uffelen J, Salmon J (2015). Total and domain-specific sitting time among employees in desk-based work settings in Australia. Aust N Z J Public Health.

[26] Owen N, Sugiyama T, Eakin E, Gardiner PA, Tremblay MS, Sallis JF (2011). Adults’ sedentary behaviour. Determinants and interventions. Am J Prev Med.

[27] Rachlin H (1989). Judgement, decision and choice: a cognitive/behavioural synthesis.

[28] McLeroy KR, Bibeau D, Steckler A, Glanz K (1988). An ecological perspective on health promotion programs. Health Educ Q.

